# NK Cell Exhaustion

**DOI:** 10.3389/fimmu.2017.00760

**Published:** 2017-06-28

**Authors:** Jiacheng Bi, Zhigang Tian

**Affiliations:** ^1^Shenzhen Laboratory of Antibody Engineering, Institute of Biomedicine and Biotechnology, Shenzhen Institutes of Advanced Technology, Chinese Academy of Sciences, Shenzhen, China; ^2^School of Life Sciences and Medical Center, Institute of Immunology, Key Laboratory of Innate Immunity and Chronic Disease of Chinese Academy of Science, University of Science and Technology of China, Hefei, China; ^3^Collaborative Innovation Center for Diagnosis and Treatment of Infectious Diseases, State Key Laboratory for Diagnosis and Treatment of Infectious Diseases, First Affiliated Hospital, College of Medicine, Zhejiang University, Hangzhou, China

**Keywords:** tumors, chronic infections, immune evasion, immune checkpoints, immunotherapy

## Abstract

Natural killer cells are important effector lymphocytes of the innate immune system, playing critical roles in antitumor and anti-infection host defense. Tumor progression or chronic infections, however, usually leads to exhaustion of NK cells, thus limiting the antitumor/infection potential of NK cells. In many tumors or chronic infections, multiple mechanisms might contribute to the exhaustion of NK cells, such as dysregulated NK cell receptors signaling, as well as suppressive effects by regulatory cells or soluble factors within the microenvironment. Better understanding of the characteristics, as well as the underlying mechanisms of NK cell exhaustion, not only should increase our understanding of the basic biology of NK cells but also could reveal novel NK cell-based antitumor/infection targets. Here, we provide an overview of our current knowledge on NK cell exhaustion in tumors, and in chronic infections.

## Introduction

Immune cell exhaustion describes the status of dysfunction of immune cells, usually under the settings of tumors or chronic infections (1, 2). Such status, usually associated with poor control of malignancies or infections, is characterized by decreased effector functions (1). For T cells, exhaustion is accompanied by phenotypic changes (1), epigenetic modifications (3), and alterations in transcriptional profiles (4). Multiple negative regulatory pathways (e.g., immunoregulatory cytokines and PD-1) have been shown to be involved in the exhaustion of T cells (1). The in-depth descriptions of the molecular characteristics of T cell exhaustion have not only provided a framework for better understanding T cell biology in these contexts but have also given rise to T cell-based antitumor or anti-infection immunotherapy, which includes immune checkpoint blockade (5) and adoptive T cell therapy (6, 7).

NK cells, as a critical part of the innate immune system, are an important effector lymphocyte population in antitumor and anti-infection immunity (8–10). Evidence supporting its essential roles includes the correlation of poor cytotoxicity of NK cells in the peripheral blood with higher risk of cancer (11). Also, the expression of NKp30 and NKG2D on NK cells from melanoma metastatic lymph node (M-LN) negatively correlated with percentages of tumor cells in M-LN (12). Not only the potentials of NK cells in controlling blood cancers and tumor metastasis have already been widely appreciated but the tumor infiltration of NK cells was also associated with good prognosis in multiple solid tumors (13–17).

However, under the settings of tumors and chronic infections, NK cells exhibit an exhausted status similar with exhausted T cells, displaying poor effector function and altered phenotype. Although the exact mechanisms leading to NK cell exhaustion in tumors and chronic infections are poorly defined, emerging studies to be discussed below have shown that multiple negative regulatory pathways in these contexts might contribute to such exhausted status of NK cells, such as dysregulated NK cell receptors signaling, as well as suppressive effects by regulatory cells or soluble factors within the microenvironment. Here, we reviewed current understanding of the characteristics and the mechanisms of NK cell exhaustion, as well as ongoing efforts trying to reverse such state of NK cells.

## NK Cell Effector Functions

NK cells mediate antitumor or anti-infection immunity by production of effector cytokines, or by direct cytotoxic activity (8). NK cells are an early and essential source of IFN-γ *in vivo* (18). IFN-γ either directly enhances target cell immunogenicity (19) or facilitates adaptive immunity (20, 21). Besides rapid production of IFN-γ, NK cells also directly eliminate transformed cells or infected cells through cytotoxic activity dependent on perforin and granzyme (22–24), or inducing target cell apoptosis by TNF-α (25), FasL (26), and TRAIL (27). In addition to the effector functions, NK cells also potentiate adaptive immune response through DC editing and maturation (28, 29).

Unlike cytotoxic T cells, NK cells are recombinase independent, and do not need to be primed before effector functions, which makes NK cells a rapid responder in host immunity. Activation of NK cells depends on the integration of activating signals and inhibitory signals from cell surface receptors (30), upon recognition of target cells (31) or interaction with accessory cells (32). Activating receptors include NKG2D, CD16, NCRs, CD226 (DNAM-1), and 2B4, among which, CD16 plays a key role in antibody-dependent cell-mediated cytotoxicity as the Fcγ receptor. Inhibitory receptors include self-MHC I-recognizing KIRs in human or Ly49s in mice, NKG2A, TIM-3, TIGIT, and CD96.

## Characteristics of NK Cell Exhaustion

### Exhausted Effector Functions

**Figure 1:**
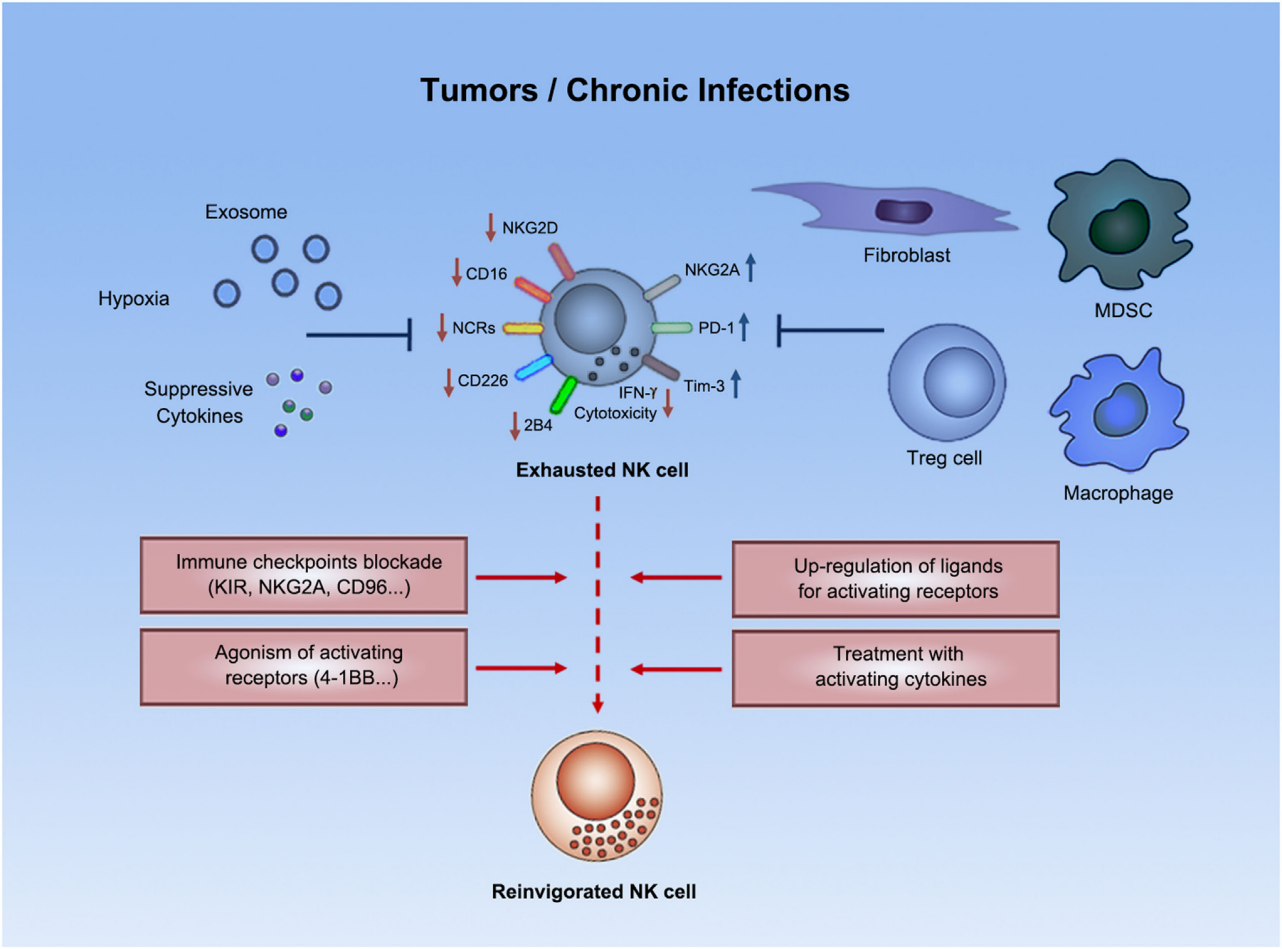
Natural killer cell exhaustion. Tumor progression or chronic infections usually leads to exhaustion of NK cells. Exhausted NK cells are characterized by decreased production of effector cytokines (e.g., IFN-γ), as well as by impaired cytolytic activity. Exhausted NK cells downregulated expression of certain activating receptors and upregulated expression of inhibitory receptors. Both suppressive cells and other suppressive factors (e.g., exosomes, suppressive cytokines, hypoxia, etc.) in tumors or chronic infections might contribute to such exhausted status. Emerging strategies (e.g., immune checkpoint blockade) could potentially reverse NK cell exhaustion to boost antitumor or anti-infection immunity.

Despite the potential cytolytic activity of NK cells against tumor cells or infected cells, NK cells exhibited impaired effector functions in hosts with tumors or chronic infections (Figure 1). For example, progression of multiple myeloma in mice was associated with decreased percentages of NK cells (33). At single cell levels, tumor-infiltrating NK cells produced decreased effector cytokines IFN-γ and GM-CSF in mouse models (34). NK cells in cancer patients showed diminished cytolytic activity, as evidenced by lower expression of cytolytic molecules, such as granzymes, perforin, FasL, and TRAIL (35). Intratumoral NK cells from patients with various cancers produced decreased IFN-γ (36, 37), CD107a (36, 37), granzyme B (36), and perforin (36) and exhibited impaired cytolytic activity (38), compared with NK cells from peritumor regions or from the peripheral blood. Such exhaustion of NK cell functions seems to be the result of an active process in tumors or chronic infections, since adoptively transferred murine NK cells into mice with leukemia rapidly lost IFN-γ production, followed by loss of cytotoxicity after homeostatic proliferation in the presence of tumor (39).

### Exhausted Phenotypes

The functional exhaustion of NK cells in tumors and chronic infections is sometimes accompanied with the downregulated expression of certain surface activating receptors on NK cells (Figure 1). NKG2D was frequently downregulated on NK cells in patients with various kinds of malignancies, e.g., pancreatic cancer, gastric cancer, colorectal cancer (35), breast cancer (38), and chronic lymphocytic leukemia (40), as well as in patients with chronic virus infection, such as HBV (41). Compromised NKG2D signaling in this context was also evidenced by downregulation of DAP10, the signaling adaptor of NKG2D (41). Besides NKG2D, CD16 (38), NCRs (NKp30, NKp44, and NKp46) (35, 38, 40–42), CD226 (33, 38, 40, 42, 43), and 2B4 (41) expression on NK cells also usually decreased under settings of tumors or chronic infections. Dysregulated expression of these receptors in patients could be restored in remission (38). Given that NK cell activation result from an integration of activating and inhibitory signals (30), weakened signals from activating receptors might result in the lost of integrated signaling balance toward domination by inhibitory signals, thus gradually inducing NK cell exhaustion.

Another phenotypic signature of NK cell exhaustion is the upregulation of inhibitory receptors (Figure 1). For example, PD-1, as a well-known target in immunotherapy, is a proven checkpoint on T cells. PD-1 overexpression in NK cell line resulted in decreased degranulation, indicating that PD-1 signaling is suppressive not only on T cells but also on NK cells (44). PD-1 was found to be upregulated on NK cells from tumor patients, such as those with Kaposi sarcoma (44), renal cell carcinoma (45), multiple myeloma (46), and EBV-associated posttransplant lymphoproliferative disorders (47). Such upregulation of PD-1 on NK cells was found confined to a subset of CD56^dim^NKG2A^−^KIR^+^CD57^+^ cells, as reported in HCMV, and ovarian carcinoma patients (48), where such subset was frequently detected (48). Such high PD-1 expression was found associated with reduced proliferative capability in response to cytokines (48), impaired degranulation (44, 48), and poor cytokine production (44, 47, 48) by NK cells. In addition, in renal cell carcinoma, increased PD-1 expression on NK cells in the peripheral blood was correlated to disease stage; the expression was significantly reduced soon after surgical resection of the primary tumor (45). Together, these data indicate that PD-1 is not only associated with the exhausted status of NK cells but PD-1 signaling also might contribute to NK cell exhaustion.

Inhibitory receptor NKG2A was increased on CD56^dim^ NK cells from hepatocellular carcinoma (HCC) patients (36). Such high expression of NKG2A, suggested to be induced by IL-10, contributed to NK cell dysfunction in these patients, correlated with plasma IL-10 levels, and predicted a poor prognosis (36). NKG2A was also expressed on higher percentages of NK cells in active chronic hepatitis B (CHB) patients, than in inactive CHB patients or healthy controls (49). In consistent with this, NK cells from HBV-carrier mice also had higher percentages of NKG2A^+^ NK cells compared with control mice (49). NKG2A was inhibitory on active-CHB patients-derived peripheral NK cell cytotoxicity (49). Importantly, NKG2A-Qa-1 interaction in HBV-carrier mice contributed to HBV persistence (49). These data suggest that inhibitory receptor NKG2A also might contribute to NK cell exhaustion in tumors and chronic infections.

Tim-3, a novel checkpoint on T cells, was shown to be an inhibitory receptor on NK cells (50) and was upregulated on NK cells from PBMCs of patients with CHB infection (51). Tim-3 suppressed the cytotoxicity of NK cells from these patients (51), suggesting that Tim-3 might be involved in the exhausted cytolytic function of NK cells in these patients.

### Exhausted Signaling/Transcriptional Programs

The maintenance of NK cell function relies on an active signaling/transcriptional program, which seems to be perturbed in tumors or chronic infections, thus contributing to NK cell exhaustion. IL-15 signaling was shown to be defective in tumor-bearing mice, which impeded NK cell maturation and IFN-γ production (52). Overexpression of IL-15 reversed the maturation defects in NK cells (52). Along with the exhaustion of NK cells, the transcriptional factor Eomes and T-bet were downregulated in adoptive transferred murine NK cells into mice with leukemia (39). Enforced expression of Eomes in NK cells partially reversed NK cell dysfunction (39), indicating that the repressed expression of Eomes in NK cells in leukemia is not only an exhaustion-associated transcriptional signature but also at least partially accounted for the exhaustion of NK cells.

## Immunoregulatory Pathways Involved in NK Cell Exhaustion

### Detrimental Modulations of Checkpoint Ligands

Together with the dysregulation of NK cell surface receptors, detrimental modulations of ligand expression on transformed or infected cell surface for NK cell receptors lead to aggravated inhibitory signals, and to dampened activating signals, which might lean the integrated signals balance toward inhibition, and promote the exhaustion of NK cells in tumors or chronic infections. For example, HLA-E, the ligand for inhibitory receptor NKG2A, was more frequently expressed by tumor cells than by normal cells, which aggravated NKG2A-mediated inhibition of NK cell activity (53). CD200, a ligand of NK cell inhibitory receptor CD200R, is upregulated in acute myeloid leukemia and is associated with poor patient outcome (54). Galactin-9, the ligand for human NK cell inhibitory receptor Tim-3 (55), was expressed at high levels on acute myeloid leukemia cells (56). In these examples, upregulated ligand for inhibitory receptor aggravated the inhibitory signaling. Whereas CD48, the ligand for activating receptor 2B4, was reported to be downregulated by oncogenic proteins in acute myeloid leukemia, thus weakening activating signaling from 2B4 (57). Presence of soluble ligand of activating receptors could also weaken activating signaling. Activating receptor GITR ligand (sGITRL) was detected in the sera of patients with various tumors (58). sGITRL or patients’ sera that contained sGITRL might block activating receptor GITR interaction with its ligand and were suppressive on NK cell cytotoxicity by negatively regulating NF-κB activity (58). Faq2 protein of *Fusobacterium nucleatum*, bacteria present in the tumor microenvironment, is a ligand for inhibitory receptor TIGIT (59–63). Faq2 was found able to bind to tumor cells and to mediate inhibition of NK cell activity by triggering TIGIT signaling (59). The abovementioned examples, either aggravating inhibitory signaling or dampening activating signaling, might promote NK cell exhaustion within tumor environment.

Upregulated ligands for NK cell activating receptors by tumor cells, in some cases, could be paradoxically inhibitory on NK cell function, possibly contributing to NK cell exhaustion as well. MICA/B, ligands for human NKG2D, was frequently expressed in both solid tumors (64) and leukemia (65). NK cells from patients with acute myeloid leukemia patients showed downregulated expression of activating receptors, NKp46, NKp30, CD226, 2B4, and CD94/NKG2C (65). Soluble MICA/B also downregulated NKG2D expression on an NK cell line *in vitro* (64), suggesting that downregulation of activating receptors on NK cells might be a result of chronic exposure of NK cells to tumor cells (65). Similarly, CD112, the ligand for both activating receptor CD226 and inhibitory receptor TIGIT in humans, is frequently expressed at high levels on tumor cells (65). Coculture with CD112-expressing leukemic cells *in vitro* was shown to downregulate CD226 on NK cells (43). In line with this, CD112 expression on leukemic blasts was negatively correlated with CD226 expression on NK cells (43). On the other hand, Rae-1, the murine ligand for NKG2D, when expressed by RMA cells, was reported to be acquired by NK cells, which elicited NK cell fratricide by neighboring NK cells through the NKG2D-induced perforin pathway both *in vitro* and *in vivo* (66). In the cases demonstrated above, upregulated activating ligands, either downregulated expression of activating receptor after NK cell chronic exposure to tumor cells or induced NK cell fratricide, ultimately leading to weakened signaling through activating receptors, which, together with other negative regulatory pathways in tumor microenvironment, might lead to NK cell exhaustion.

Posttranslational alterations of ligands for NK cell surface receptors might also be involved in NK cell exhaustion. For example, posttranslational modifications reduced the affinity of MICA for NKG2D, impairing activating signals from NKG2D (67). Diminished activating signaling might then gradually induce the exhausted status of NK cells in tumor microenvironment.

### Regulatory Cells

In addition to modulations of NK cell surface receptor signaling, suppressive immune cells and non-immune cells exist in the microenvironment of tumors or chronic infections, which rendered exhaustion of NK cells (Figure 1).

CD4^+^CD25^+^Foxp3^+^ regulatory T cells are a critical subset of T cells that maintain immune tolerance (68–70). Tumor growth or chronic infections promotes the expansion of Tregs (71–73). Treg cells are inhibitory on NKG2D expression on NK cells, and on NK cell cytotoxicity (74). Evidence that Treg cells are related to NK cell exhaustion is that absolute numbers of Treg cells in patients with gastrointestinal stromal tumors inversely correlated with NK cell induction (74).

Tumor growth also promotes the expansion of another suppressive cell type, CD11b^+^Gr1^+^ myeloid-derived suppressor cells (MDSCs) (75). Expansion of MDSCs inversely correlated with activation of NK cells in tumor patients and mice (76), linking MDSCs with NK cell exhaustion. MDSCs suppressed IL-2-mediated NK cell cytotoxicity by affecting the activity of STAT5 in a contact-dependent manner (77). HCV-induced MDSCs suppressed NK cell IFN-γ production *via* an arginase-1-dependent inhibition of mechanistic target of rapamycin (mTOR) activation (78). MDSCs inhibited NKG2D expression on NK cells and suppressed both cytotoxicity and IFN-γ production, both *in vitro* and *in vivo via* membrane-bound TGF-β in mice (76). These data indicate that MDSCs could mediate NK cell exhaustion in tumors and chronic infections through their suppressive functions.

Besides CD11b^+^Gr1^+^ cells, the tumor promoting roles of other myeloid cells (79, 80) have been well accepted, which are potentially suppressive on NK cell functions in tumors as well. For example, in human gastric cancers, tumor-infiltrating monocytes/macrophages were physically close to NK cells, and their percentages were negatively correlated with the percentages of IFN-γ^+^ and TNF-α^+^ cells among NK cells (16), indicating that these tumor-infiltrating monocytes/macrophages are associated with NK cell exhaustion. Such monocytes/macrophages could impair expression of IFN-γ, TNF-α, and Ki-67 in NK cells in a TGF-β-dependent manner *in vitro* (16), suggesting that these monocytes/macrophages might contribute to the exhaustion of NK cells in tumors.

On the other hand, increasing evidence indicated the NK-suppressive roles of non-immune cells in tumors. Fibroblasts derived from metastatic melanomas mediated inhibition of IL-2-induced upregulation of CD226 on NK cells in a contact-dependent manner and counteracted IL-2-induced upregulation of NKp44 and NKp30 through releasing PGE(2) (81). Fibroblasts from HCC were significantly superior to foreskin-derived fibroblasts at impairing NK cell activation, which was mediated by PGE(2) and IDO (82). However, the contribution of tumor-derived fibroblasts to NK cell exhaustion in physiological conditions still needs further investigations.

### Exosomes

Tumor-associated exosomes are immunoregulatory microvesicles secreted from tumor cells (Figure 1). Breast tumor cell-derived exosomes inhibited perforin expression and cytolytic activity *ex vivo* and *in vitro* and reduced NK cell percentages in both the lung and spleen of naive mice (83). Tumor cell-derived exosomes suppressed NK cell function by expressing TGF-β, and by expressing ligands for NKG2D to downregulating NKG2D expressed by NK cells (84). Exosomes from the sera of acute myeloid leukemia patients also showed NK-suppressive effects by similar mechanisms (85). Moreover, exosomes from hypoxic tumors showed TGF-β1 and miR-210- and miR-23a-dependent suppression on NK cell function, as compared with exosomes from normoxic tumor cells, in multiple tumor models (86). These studies demonstrated the inhibitory effects of tumor-associated exosomes on NK cells and suggest that they might be involved in the exhausted status of NK cells in these contexts.

### Suppressive Cytokines

Suppressive cytokines are important factors that might promote NK cell exhaustion in tumors and in chronic infections (Figure 1). TGF-β was usually detected at high levels in the settings of tumors (87) or chronic infections (41), indicating that TGF-β is highly associated with tumors and chronic infections. Besides, inhibitory effects of TGF-β on NK cells are well documented. Expression of NKG2D was shown to be downregulated by TGF-β (88). TGF-β also induced miR-183 to repress DAP12 transcription, thus suppressing signaling of activating NK cell receptors (89). TGF-β-treated human NK cells exhibited decreased cytolytic activity, with abrogated perforin polarization to the immune synapse (89). Breast tumor cell-secreted TGF-β suppressed NK cell expression of activation marker CD69, degranulation marker CD107a, effector cytokines IFN-γ and TNF-α, and cytotoxicity against target cells (38). Despite these *in vitro* studies and correlation analyses, the exact contributions of TGF-β to NK cell exhaustion in physiological conditions have yet to be revealed.

### Hypoxia

Other characteristics of tumor microenvironment might contribute to NK cell exhaustion, e.g., hypoxia (Figure 1). Within tumor microenvironment, oxygen is usually less available than in normal tissues, which has been long associated with the immune suppressive characteristics of tumors (90). Hypoxia has been shown to induce NK cell suppression. Hypoxia induced hypoxia-inducible factor 1α in NK cells, decreased expression of NKG2D expression (91), and abrogated the upregulation of NKp46, NKp30, NKp44, and NKG2D in response to activating cytokines (92), thus impairing the capacity of killing target cells (92). Future studies are required to elucidate the role of hypoxia in NK cell exhaustion *in vivo*.

## Reversion of Exhaustion

Blockade of immune checkpoints, as an important part of immunotherapy, proved effective in reversing the exhaustion of T cells to boost antitumor immunity. However, in immunotherapy for tumors and chronic infections, treatment with anti-PD-1 monoclonal antibody alone or with an additional anti-CTLA-4 monoclonal antibody has exhibited clinical benefits only for some patients, suggesting the need for combining extra therapeutics that further counteract the immuno-suppresive mechanisms by tumors or infected cells. In this context, mechanisms that mediate NK cell exhaustion in chronic disorders, as well as pathways for maintaining NK cell self-tolerance, may be targeted to reinvigorate NK cells and provide additionally enhanced immunity (Figure 1).

### Checkpoint Blockades/Agonisms

First of all, emerging checkpoint blockade strategies are being tested for the potential in reversing NK cell exhaustion in tumors and chronic infections. While blocking KIR, in combination with CTLA-4 blockade, is still under clinical trials for treatment of advanced tumors (NCT01750580), preclinical studies have shown that KIR (or Ly49I/C in mice) blockade boosted NK cell activity in tumor models. Treatment of leukemia-bearing mice with F(ab')_2_ of a blocking antibody against mouse Ly49I/C or adoptive transfer of NK cells treated *ex vivo* with F(ab')_2_ increased the survival rate (93). F(ab')_2_ of anti-Ly49I/C also enhanced the antileukemia activity of anti-huCD20 monoclonal antibody in an EL4-huCD20 leukemia mouse model in an NK-dependent manner (94). A fully human monoclonal antibody, lirilumab, reacts with KIR2DL1/2/3, preventing their binding to HLA-C (95). Administration of lirilumab enhanced the beneficial effect of anti-huCD20 monoclonal antibody, rituximab, on mouse survival in the EL4-huCD20 leukemia model in Rag1KO-KIR Tg mice, whose effect was abrogated when NK cells were depleted (94). Anti-KIR monoclonal antibody and lenalidomide also combined to enhance NK cell versus multiple myeloma effect (96).

Besides KIR, preclinical studies have been revealing more potential NK cell checkpoints. Therapeutic CD94/NKG2A blockade with an anti-NKG2A monoclonal blocking antibody, monalizumab, restored direct cytotoxicity of NK cells against chronic lymphoid leukemia cells *in vitro* (97). Blocking NKG2A-Qa-1 interaction *in vivo* in HBV-carrier mice promoted viral clearance in an NK cell-dependent manner (49). CD96, together with activating receptor CD226 and inhibitory receptor TIGIT, constitutes a receptor family that bind nectins and nectin-like family proteins (e.g., CD155 and CD112) and regulates NK cell functions (98). CD96, previously shown to promote human NK cell–target cell adhesion (99), was later revealed to compete with CD226 for CD155 binding and directly inhibits IFN-γ production by NK cells in mice (100). CD96^−/−^ mice displayed resistance to carcinogenesis and experimental lung metastasis (100). Blocking CD96 with a monoclonal antibody inhibited experimental metastases in multiple mouse models (101).

Agonist antibody to augment activating signaling for NK cells is another potent strategy to boost NK cell activity. Administration of anti-4-1BB agonist monoclonal antibody enhanced the expression of CD69 activation marker on NK cells and improved disease control in an NK cell-dependent manner in mice with established multiple myeloma (33).

### Checkpoint Modulations

Besides checkpoint inhibitors and agonists, emerging agents potentially reverse NK cell exhaustion by counteracting the detrimental regulation of NK cell receptors or their ligands in the microenvironment of tumors or chronic infections. For example, tumor cell surface expression of NKG2D ligands, either in human or in mice, could be therapeutically upregulated to enhance NK cell activation, by treating tumor cells with alkylating agent (102), proteasome inhibitors (103), hyperploidy-inducing agents (104), histone deacetylation inhibitors (105), or inhibitors for glycogen synthase kinase-3 (106). On the other hand, tumor surface expression of MHC class I might also be downmodulated to increase miss-self recognition-mediated activation of NK cells, e.g., by proteasome inhibitors (107) or PI3K inhibitors (108).

### Cytokines

In addition to NK cell surface receptor signaling–targeting/modulating agents, treatment with activating cytokines or blocking the signaling of suppressive cytokines might reverse the NK-disfavoring cytokine milieu that might promote NK cell exhaustion in tumors and chronic infections. Treatment with NK-activating cytokines, IL-12 and IL-18, or with an IL-2 mutant (H9 “superkine”) increased survival of MHC-I-deficient tumor-bearing mice, accompanied by restoration of effector functions of MHC-I-deficient tumor-infiltrating NK cells (109). Alternatively, another NK-activating cytokine, IL-15 was under clinical trials for treatment of various tumors. In addition, IL-15 fused with the extracellular domain of NKG2D was shown to exhibit enhanced NK cell tumor infiltration and increased suppression of xenografted tumors growth in nude mice infused with human PBMC (110). On the other hand, blockade of TGFβR1 with a small molecule Galunisertib restored TGF-β-induced downregulation of NK cell activating receptors, CD226, NKp30, and NKG2D, and cytolytic molecules, TRAIL, perforin, and granzyme A, increasing direct cytotoxicity and ADCC of *ex vivo* activated NK cells against neuroblastoma cells *in vitro* (111).

## Remaining Questions and Future Directions

In many tumors and chronic infections, the potential of NK cells to produce cytotoxic molecules and effector cytokines is restricted, accompanied with detrimentally modulated expression of surface receptors/ligands, indicating that, similar with T cells, NK cells are also exhausted in these settings. Based on our limited knowledge about NK cell exhaustion, there have been attempts trying to reverse NK cell exhaustion to boost antitumor or anti-infection immunity. However, current barriers in reversing NK cell exhaustion lie beyond the lack of in-depth understanding of such status.

Recent studies suggest that exhausted CD8^+^ T cells in many tumors and chronic infections may represent a distinct lineage, in that exhausted T cells display multiple signatures distinct with effector T cells and memory T cells (1, 3). On the other hand, the molecular basis of exhausted NK cell in tumors and chronic infections remains largely unexplored.

One feature of exhausted CD8^+^ T cells in tumors and chronic infections is that transcriptional pathways are used differently by exhausted CD8^+^ T cells than by effector and memory CD8^+^ T cells. For example, Blimp-1 is expressed at aberrantly high levels by exhausted CD8^+^ T cells during chronic infections, which promotes the expression of inhibitory receptors and exhaustion (112). Although the transcriptional factors specific for exhausted NK cells are yet to be defined, Eomes and T-bet, essential for the effector function of NK cells (113), were shown to be downregulated in exhausted NK cells in leukemia (39). Overexpression of Eomes restored the exhausted function of NK cells, indicating that downregulated Eomes accounted for the exhausted phenotype of NK cells in this context (39).

Besides transcriptional regulations, cellular metabolism is also important for lymphocyte development and effector function (114–116). Through comparing CD8^+^ T cells in either chronic or acute infections, the exhaustion of CD8^+^ T cells in chronic infections was linked to suppressed bioenergetics, despite persistent signaling of mechanistic target of rapamycin (mTOR) pathway (117). Since activation of mTOR pathway is critical for the effector function of NK cells (118), the exhausted function of NK cells suggests that this pathway in NK cells might be altered in tumors and chronic infections, which remains to be determined.

Emerging evidence appreciates the role of epigenetic regulation in immune cell differentiation and function (119). Exhausted CD8^+^ T cells exhibited stable and state-specific epigenetic modifications distinct from functional memory CD8^+^ T cells (120, 121). The histone-lysine N-methyltransferase, enhancer of zeste homolog 2 (EZH2), was shown to regulate NK cell differentiation and function (122), indicating that epigenetic regulation plays a critical role in shaping NK cell response. However, the epigenetic signature of exhausted NK cells in tumors and chronic infections has not been revealed.

Taken together, the molecular basis, such as transcriptional profiles, epigenetic state, and metabolic regulation, of exhausted NK cells in tumors and chronic infections, as well as the clinical relevance, is still elusive. Future studies are required to reveal novel mechanisms/pathways, e.g., in the aspects mentioned above, hopefully leading to novel targets for NK-based immunotherapy.

## Author Contributions

Both JB and ZT conceived and wrote the manuscript.

## Conflict of Interest Statement

The authors declare that the research was conducted in the absence of any commercial or financial relationships that could be construed as a potential conflict of interest.
